# Novel Hydrophobin Fusion Tags for Plant-Produced Fusion Proteins

**DOI:** 10.1371/journal.pone.0164032

**Published:** 2016-10-05

**Authors:** Lauri Reuter, Anneli Ritala, Markus Linder, Jussi Joensuu

**Affiliations:** 1 VTT Technical Research Centre of Finland Ltd., Espoo, Finland; 2 Aalto University, Department of Biotechnology and Chemical Technology, Espoo, Finland; Austrian Institute of Technology, AUSTRIA

## Abstract

Hydrophobin fusion technology has been applied in the expression of several recombinant proteins in plants. Until now, the technology has relied exclusively on the *Trichoderma reesei* hydrophobin HFBI. We screened eight novel hydrophobin tags, *T*. *reesei* HFBII, HFBIII, HFBIV, HFBV, HFBVI and *Fusarium verticillioides* derived HYD3, HYD4 and HYD5, for production of fusion proteins in plants and purification by two-phase separation. To study the properties of the hydrophobins, we used N-terminal and C-terminal GFP as a fusion partner. Transient expression of the hydrophobin fusions in *Nicotiana benthamiana* revealed large variability in accumulation levels, which was also reflected in formation of protein bodies. In two-phase separations, only HFBII and HFBIV were able to concentrate GFP into the surfactant phase from a plant extract. The separation efficiency of both tags was comparable to HFBI. When the accumulation was tested side by side, HFBII-GFP gave a better yield than HFBI-GFP, while the yield of HFBIV-GFP remained lower. Thus we present here two alternatives for HFBI as functional fusion tags for plant-based protein production and first step purification.

## Introduction

Hydrophobins (HFB) are small, secretory proteins found in filamentous fungi with diverse biological functions [1,2]. The compact globular structure is stabilized by four disulphide bonds between conserved cysteine residues. A hydrophobic patch, exposed on the surface of the protein, gives HFBs their hydrophobic and extraordinarily surface active properties [1]: They assemble in aqueous solutions, interact with non-ionic surfactants and self-assemble into monolayers at liquid-air interfaces and on hydrophobic surfaces [3,4]. HFBs are divided into classes I and II based on differences in their hydrophobicity plots, solubility and spacing of the conserved cysteine residues [1,2].

The HFBs have several applications in biotechnology ranging from food additives [5] to functional coatings in nanomedicine [6]. When used as fusion partners for recombinant proteins, HFBs convey some of their functionalities to the respective fusion protein. This approach has been applied e.g. in immobilization of bioactive proteins on biosensors [7] or recruiting cellulose nano-fibrils into films to air-water or oil-water interfaces [3]. HFB fusion technology has been further applied to purification of recombinant proteins using aqueous two-phase separation (ATPS)[8,9]. ATPS is a low-cost and scalable method for first step purification of recombinant proteins in fungal [8,9], insect [10], plant [11,12] and plant cell based production platforms [13].

When expressed as a fusion protein in plants or plant cell cultures, the *Trichoderma reesei* hydrophobin I (HFBI)[14] induces formation of protein bodies (PB) [12,13,15,16]. PBs are dense, spherical structures derived from the endoplasmic reticulum (ER). The mechanism of PB formation remains unclear, but it is thought to relate to the self-assembly and interaction of the fusion proteins in the ER. HFBI as a fusion tag improves accumulation of GFP in plants significantly [12,15] and has also increased the yield of some other target proteins [17], but not all [18,19]. The use of HFBI and other PB inducing tags has been reviewed earlier [20].

**Fig 1:**
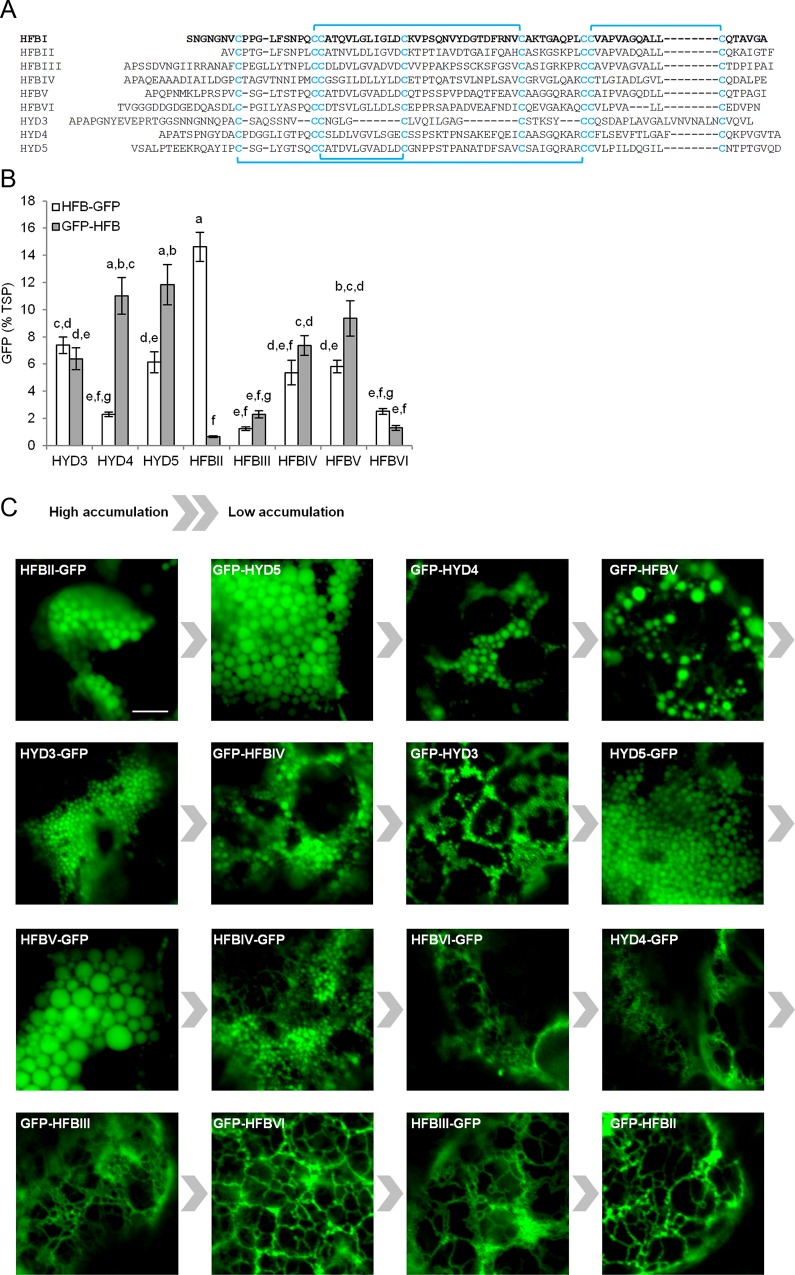
Expression of the HFB library in *N*. *benthamiana*. (A) Amino acid sequences of the HFBI and the 8 novel hydrophobin fusion tags studied here. Conserved cysteine residues and disulphide bridges are highlighted. (B) Expression levels of fusion proteins determined by fluorometry. Letters indicate groups with significant difference (*p*<0.05, *n* = 8 individual plants). Error bars indicate standard deviation. (C) Confocal microscopy images illustrate subcellular localization of the fusion proteins. A control sample infiltrated with only p19 showed no signal (image not show). The scale bar represents 5μm.

Thus far, several HFB fusion proteins have been expressed in plants [12,16–19,21]. However the application of HFB fusion technology in plants has relied solely on HFBI, a class II HFB. Aside from the conserved cysteine residues (Fig 1A), HFBs share little homology in amino acid sequences [1,2]. The genes coding for HFBs are found in small families and they are expressed differentially, both spatially and temporally [1,22,23]. This indicates that HFBs may have different roles throughout the fungal lifecycle and therefore also different functional properties. To explore the diversity of other HFBs as potential fusion tags, we have created a library of eight HFBs fused to both termini of GFP. The library covers rest of the characterized HFBs of *T*. *reesei*, HFBII [22], HFBIII [24], HFBIV [25], HFBV and HFBVI and additionally HYD3, HYD4 and HYD5 from *Fusarium verticillioides* [23] (Fig 1A). While HYD3 is a class I HFB, all others belong to the class II. In this study, we transiently expressed the HFB library in *Nicotiana benthamiana* to evaluate the accumulation levels and tested the applicability of the novel fusion tags for protein purification through ATPS.

## Materials and Methods

### Cloning

Sequences coding for HFBs (S1 Table) without native signal sequence were codon optimized and ordered from Genscript (S2 Fig). The coding sequences for HFBs, a GS-linker (amino acid sequence: GGGSGGGSGGGSENLYFQG) and eGFP (Uniprot: A0A076FL24) were assembled in the pJJJ178 vector (S3 Fig). The HFBI-GFP, HFBII-GFP and HFBIV-GFP constructs for comparison with HFBI-GFP contained another linker (amino acid sequence: GAGGGSGGGSGGGSA). Expression vectors were introduced to *Agrobacterium tumefaciens* strain EHA105.

### Protein expression and extraction

Transient expression was done as described earlier [12]. In brief, the optical density of *A*. *tumefaciens* cultures were adjusted to 1.0 and the suspension was mixed (1:1) with Agrobacterium carrying an expression vector for post transcriptional gene silencing inhibitor p19 [26]. Agro-infiltrated *N*. *benthamiana* leaves from 8 plants were harvested 6 dpi and homogenized with extraction buffer (6:1 v/w; PBS [12 mM Na_2_HPO_4_2H_2_O, 3 mM NaH_2_PO_4_H_2_O, 150 mM NaCl), 1 mM EDTA, pH 7.4]). The extract was clarified by centrifugation (Eppendorf Centrifuge 5424R, 21130 g, 10 min, 4°C for expression analysis or Eppendorf Centrifuge 5810R, 3220 g, 10 min, RT for ATPS). Samples were analysed by fluorometry [13], for total soluble protein (TSP) by Bradford analysis (Bio-Rad, USA) and on SDS-PAGE.

### Aqueous two phase separation (ATPS)

ATPS was performed as described before [13] in 8 ml volume at RT with 4% Triton X-114 (w/v; Sigma-Aldrich). To recover the fusion proteins, the surfactant phase was extracted with 3.2 ml isobutanol (Merck). For the second round of ATPS, comparing HFBI-GFP, HFBII-GFP and HFBIV-GFP, purified (ATPS and a Strep-Tactin® column [IBA, Germany]) proteins were added to PBS to a concentration of 30 μg/ml. ATPS was performed in 1.5 ml volume with 3% Triton X-114 and recovered with 0.45 ml isobutanol. The partition coefficient (*k*) was calculated by dividing the protein concentration in the surfactant phase (protein amount in recovered phase divided by volume of the surfactant phase) by the concentration in the residue [9].

### Confocal microscopy

Localization of GFP-fusion proteins was visualized in leaf disks harvested 7 dpi using a Zeiss LSM 710 laser scanning confocal microscope (Carl Zeiss, Oberkochen, Germany) with a 63× water immersion objective (excitation at 488-nm and detection at 493–598 nm).

### Statistical analysis

Statistical analyses were done with SPSS Statistic 22.0 (IBM, Armonk, NY). A one way ANOVA test, followed by the Tukey HSD test were performed, with a significance level of 95%.

## Results and Discussion

### Accumulation of GFP and protein body formation are dependent on the HFB-fusion tag

To compare the accumulation levels, we transiently expressed GFP fused to eight different HFBs (Fig 1A), in both orientations, in *N*. *benthamiana*. Accumulation of GFP was quantified according to the fluorescence signal (Fig 1B). These results are in agreement with an SDS-PAGE analysis (S4 Fig).

The HFBII-GFP gave a yield of up to 15% GFP of TSP, but curiously the GFP-HFBII gave a yield of only less than 1%. We have observed the same trend with other target proteins fused to HFBII (unpublished data). The low accumulation of GFP-HFBII could be related to the short N-terminal sequence before the first cysteine residue (Fig 1A), which, with the linker used here, may not have been sufficient to provide space for proper folding. In addition to HFBII, the differences in accumulation levels between C-and N-terminal fusions were significant (*p*<0.05) with HYD4 and HYD5.

To investigate whether the novel fusion tags induce formation of PBs, as described earlier with GFP-HFBI [12,13,15,16], we examined the leaves expressing the proteins under confocal microscope (Fig 1C). Consistent formation of PBs, similar to GFP-HFBI [12,15,16], was apparent only with HFB-fusions with relatively high accumulation levels. Fusion proteins with lower yields, such as GFP-HFBII and both HFBIII and HFBVI fusion proteins were observed predominantly in reticulate ER. These observations are in agreement with earlier reports indicating that a threshold level of accumulation is essential for PB formation [15] and that higher concentration of protein in the ER correlates with larger and more consistent PBs [16]. It remains unclear whether the formation of PBs is a result of high concentration of protein, or a causing factor of it.

### Only fusion proteins with HFBII and HFBIV show partitioning in ATPS

**Fig 2:**
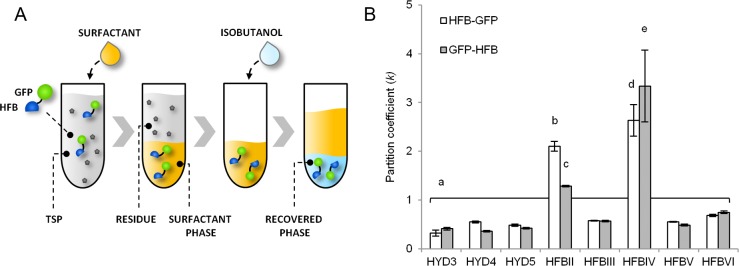
Purification of HFB-fusion proteins by ATPS. (A) A schematic illustration of the process. (B) Purification from plant extract. The partition coefficient is determined by dividing concentration in surfactant phase by concentration in residue. Letters indicate significant difference (*n* = 4, *p*<0.05) and error bars indicate standard deviation.

We set up ATPS experiments to study the performance of the HFB tags for protein purification. First we examined whether the HFB fusion proteins would separate from leaf extract (TSP) into a surfactant phase and further to the recovered fraction (Fig 2A). The partition coefficient (*k*) [9] describes the ratio of the protein concentration between surfactant phase and residue (Fig 2B).

Most fusion proteins show low partitioning to the surfactant (*k*<1) with no significant differences between the constructs. Only HFBII and HFBIV concentrate the fusion proteins into the surfactant phase (Fig 2 and S5 Fig). The *k*-values for HFBII-GFP and GFP-HFBII were 2.1±0.1 and 1.3±0.0, respectively, and for HFBIV-GFP and GFP-HFBIV 2.6±0.3 and 3.3±0.7 (mean±SD, *n* = 3), respectively. Although not functional in ATPS, other HFBs may turn out useful for other applications, such as surface adhesion. This was, however, out of our scope here.

### Efficient separation does not correlate with high accumulation

The ability of the HFB fusion proteins to interact with the non-ionic surfactant (Fig 2B), does not correlate (R^2^ = 0.0168, S6 Fig) with accumulation levels (Fig 1B). Thus the expression level of a given HFB fusion protein, or tendency to accumulate in PBs, cannot be used to predict functionality in ATPS. The characteristics enabling HFBs to interact with surfactants and/or to induce formation of PBs are not well known. However, these results indicate that the fundamental properties responsible for the two phenomena are not the same.

### Comparison to HFBI

**Fig 3:**
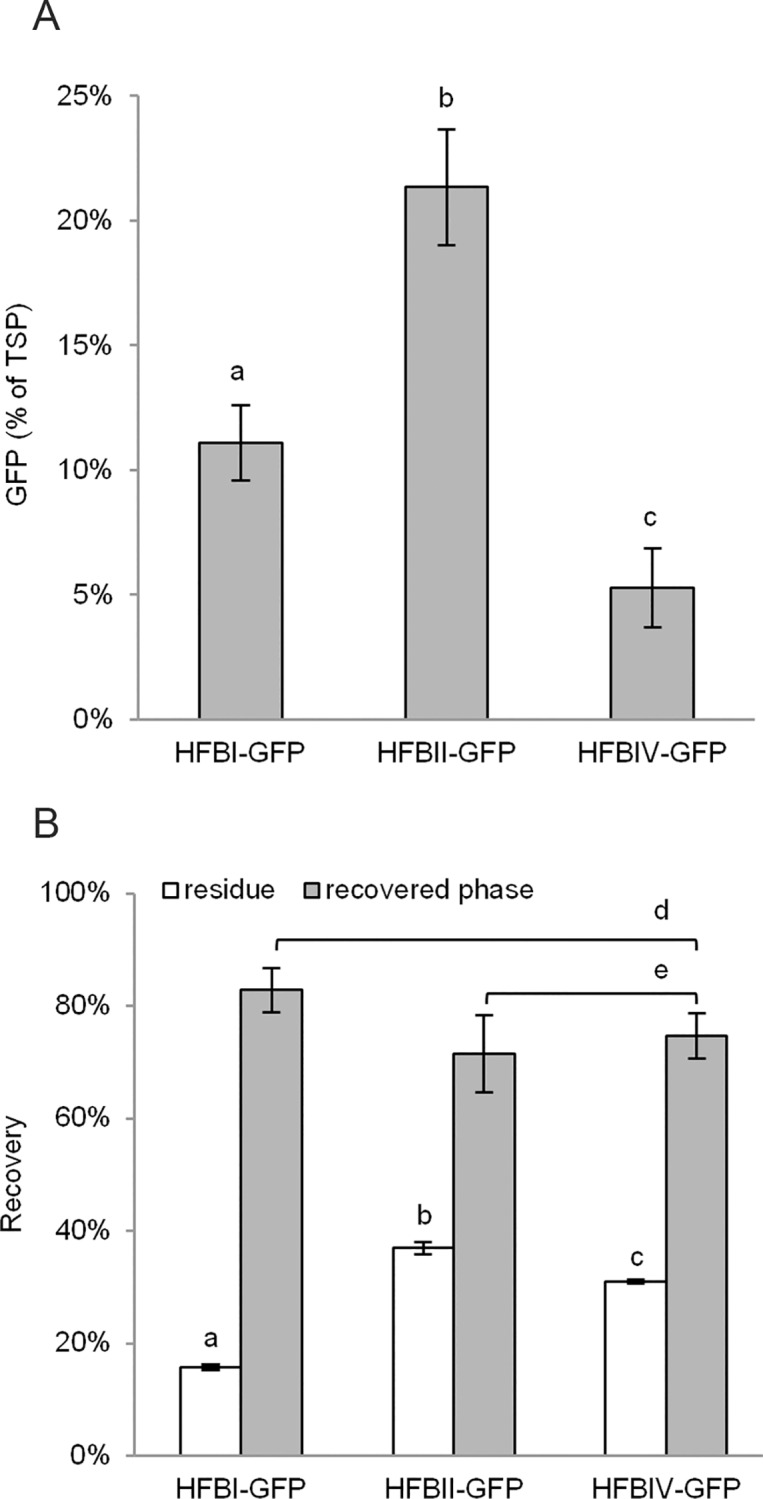
Comparison of HFBII and HFBIV to HFBI. (A) Accumulation in *N*. *benthamiana* (*n* = 8 individual plants). (B) ATPS with purified proteins to compare recovery rates (*n* = 3). Letters indicate significant difference (*p*<0.05). Error bars indicate standard deviation.

Finally we compared HFBII and HFBIV side by side with HFBI (Fig 3). We used only N-terminally fused HFBs due to the low yield of GFP-HFBII.

The yields of HFBII-GFP and HFBIV-GFP (Fig 3A) were in line with the initial screening (Fig 1B). The yield of HFBI-GFP reached only 11.1±1.5% GFP of TSP (mean±SD, *n* = 8), which is significantly less than HFBII-GFP (21.3±2.3%), but more than HFBIV-GFP (*p*<0.05). The yield of HFBI-GFP here was much lower than the yield of 38% GFP of TSP that has been previously reported for the non-codon optimized and differently oriented GFP-HFBI [12].

Next, we set up an ATPS experiment (Fig 3B) by adding purified fusion proteins into buffer in equal concentrations, because the protein concentration and the matrix of the plant extract may influence the separation efficiency [12]. The recovery yield of HFBI-GFP was 83±4% in the surfactant phase giving a *k*-value of 15.7±0.6 (mean±SD, *n* = 3). This is comparable with previous findings [12,13]. The recovery yields of HFBII-GFP and HFBIV-GFP were only slightly lower, 71±7% and 75±4%, respectively. However, the *k*-values for HFBII-GFP 5.7±0.5 (mean±SD, *n* = 3) and HFBIV-GFP 7.4±0.5 indicate significantly less efficient (*p*<0.05) separation. The slightly larger portions of the HFBII and HFBIV fusion proteins remaining in the residue were also visible on immunoblot (S6 Fig). The larger *k*-values in the later ATPS experiment reflect the smaller amount of surfactant used (3% vs. 4%) [12]. The difference in *k*-values between HFBII-GFP and HFBIV-GFP was significant (*p*<0.05), but there was no significant difference in the recovery yields. Similar behaviour of structurally similar HFBI and HFBII in the two phase system was expected based on previous reports [9]. However, the amino acid sequence and hydropathy profile of HFBIV are very different from HFBI and HFBII. In addition, most of the differences occur on the surface of the protein presumably influencing its properties [25]. To our knowledge this is the first report on separation of HFBIV in a two phase system based on a non-ionic surfactant. The two-phase separation method has been optimized for HFBI [12] and thus further optimization with other HFB´s may balance out the observed differences.

## Conclusions

Until now, only HFBI has been applied in HFB-fusion technology in plants. In this study we screened eight novel HFBs for plant based-production of fusion proteins. Accumulation of most fusion proteins remained modest, only HFBII-GFP reaching similar or even higher yields than HFBI-GFP. With some HFBs, such as HFBII, the orientation of the fusion had a significant impact on the yield. In ATPS both HFBII and HFBIV performed well, being only slightly less efficient than HFBI. It appears that the tendency to separate in surfactant based aqueous two phase systems is a property shared only by few HFBs. Although the results need to be confirmed with other target proteins than GFP, it can be concluded that, in addition to HFBI, at least HFBII and HFBIV are potential fusion partners for plant-based production of fusion proteins capable of interaction with non-ionic surfactants.

## Supporting Information

S1 Fig Expression cassettes and amino acid sequences. (A) A schematic presentation of the expression cassettes. Genes for HFB, linker and GFP were cloned in the vector between BsaI restriction sites using Golden gate assembly. (B) The full nucleotide and amino acid sequences of the coding region of the expression cassette for representative construct: HFBII-GFP. (C) Sequence data for HFBIII, (D) HFBIV, (E) HFBV, (F) HFBVI, (G) HYD3, (H) HYD4 and (I) HYD5.
(PDF)

S2 Fig Expression vector pJJJ178. (PDF)

S3 Fig Pooled leaf samples. (A) A Coomassie stained SDS-PAGE of pooled leaf samples (n = 8) showing accumulation of HFB fusion proteins (expected size indicated by arrows) in Nicotiana benthamiana. (B) Immunoblot analysis with anti StrepII-tag antibody indicates some degradation of the fusion proteins. Equal amounts of total soluble protein were loaded on all gels. A leaf infiltrated with only a construct for P19 was used as a negative control.
(PDF)

S4 Fig SDS-PAGE illustrating aqueous two phase separation. (A) Coomassie stained SDS-PAGE gels of representative samples from ATPS (Fig 1B). Lane numbering refers to: 1) TSP; 2) residue; 3) recovered phase. (B) Concentrations of fusion proteins in residue and recovered phase in comparison to initial concentration in plant leaf extract (TSP). Error bars indicate standard deviation (n = 4).
(PDF)

S5 Fig Protein accumulation levels (means, Fig 1B) blotted against k-values (means, Fig 2B). (PDF)

S6 Fig SDS-PAGE and immunoblot illustrating aqueous two-phase separation with purified proteins. (A) Coomassie stained SDS-PAGE and (B) an immunoblot of pooled samples (n = 3) from starting solution, residue phase and recovered phase in ATPS comparing HFBI-GFP, HFBII-GFP and HFBIV-GFP (Fig 3B). Detection was performed with anti-c-Myc tag primary antibody (rabbit, A00172, GeneScript) and a secondary antibody for IR-detection (goat anti-rabbit, IR Dye® 680RD, LI-COR Biosciences, Germany).
(PDF)

S1 Table Genbak ID numbers for original gene sequences and Uniprot ID numbers for amino acid sequences used in this study. The sequence coding for the extended N-terminal part of HFBVI, presumably a cell wall binding domain (amino acids 1–179), was not included in the coding sequence.
(PDF)
