# Supplementary material for: Novel Hydrophobin Fusion Tags for Plant-Produced Fusion Proteins
Source: PLoS One. 2016 Oct 5;11(10):e0164032. doi: 10.1371/journal.pone.0164032 (PMC5051927; doi:10.1371/journal.pone.0164032)
Supplement: S1 Fig — (A) A schematic presentation of the expression cassettes. Genes for HFB, linker and GFP were cloned in the vector between BsaI restriction sites using Golden gate assembly. (B) The full nucleotide and amino acid sequences of the coding region of the expression cassette for representative construct: HFBII-GFP. (C) Sequence data for HFBIII, (D) HFBIV, (E) HFBV, (F) HFBVI, (G) HYD3, (H) HYD4 and (I) HYD5. (PDF) [file pone.0164032.s001.pdf]

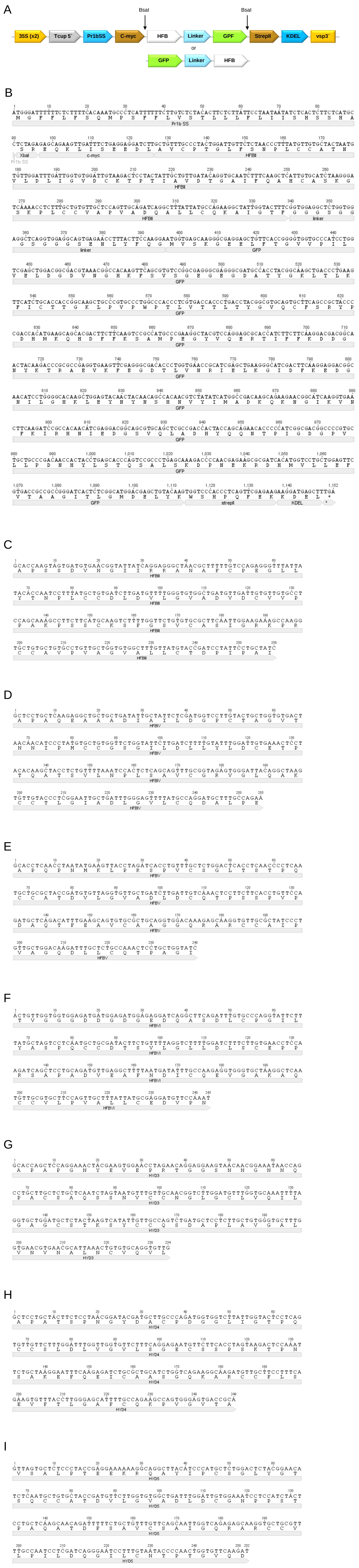

S1 Fig. Expression cassettes and amino acid sequences. (A) A schematic presentation of the expression cassettes. Genes for HFB, linker and GFP were cloned in the vector between BsaI restriction sites using Golden gate assembly. (B) The full nucleotide and amino acid sequences of the coding region of the expression cassette for representative construct: HFBII-GFP. (C) Sequence data for HFBIII, (D) HFBIV, (E) HFBV, (F) HFBVI, (G) HYD3, (H) HYD4 and (I) HYD5.
