# Supplementary figures and images for: Novel Hydrophobin Fusion Tags for Plant-Produced Fusion Proteins
Source: PLoS One. 2016 Oct 5;11(10):e0164032. doi: 10.1371/journal.pone.0164032 (PMC5051927; doi:10.1371/journal.pone.0164032)

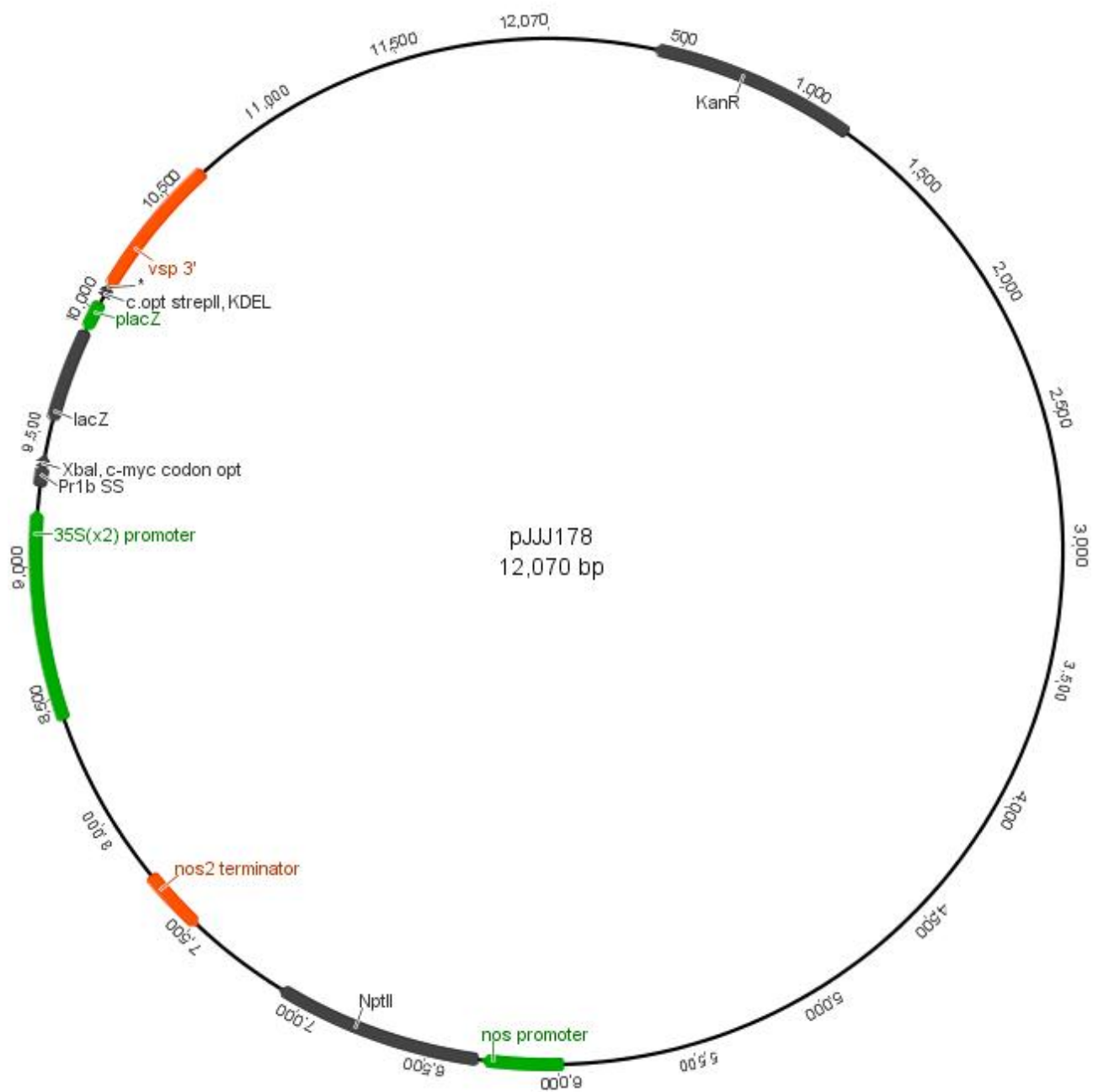

Supplement: S2 Fig — (PDF) [file pone.0164032.s002.pdf]

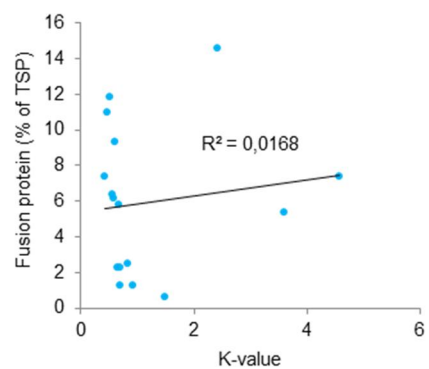

S5 Fig. Protein accumulation levels (means, Fig. 1B) blotted against k-values (means, Fig. 2B).

Supplement: S5 Fig — (PDF) [file pone.0164032.s005.pdf]
