# Supplementary material for: Novel Hydrophobin Fusion Tags for Plant-Produced Fusion Proteins
Source: PLoS One. 2016 Oct 5;11(10):e0164032. doi: 10.1371/journal.pone.0164032 (PMC5051927; doi:10.1371/journal.pone.0164032)
Supplement: S3 Fig — (A) A Coomassie stained SDS-PAGE of pooled leaf samples (n = 8) showing accumulation of HFB fusion proteins (expected size indicated by arrows) in Nicotiana benthamiana. (B) Immunoblot analysis with anti StrepII-tag antibody indicates some degradation of the fusion proteins. Equal amounts of total soluble protein were loaded on all gels. A leaf infiltrated with only a construct for P19 was used as a negative control. (PDF) [file pone.0164032.s003.pdf]

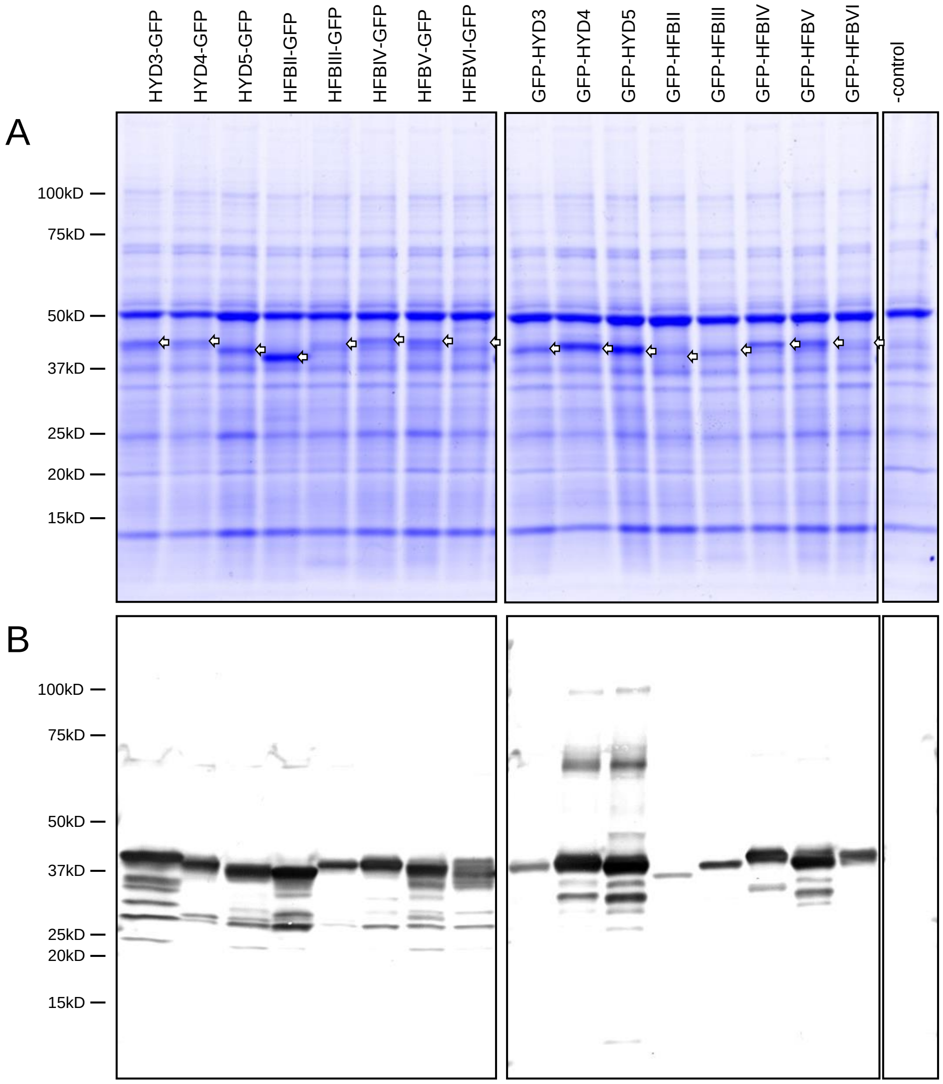

S3 Fig. Pooled leaf samples. (A) A Coomassie stained SDS-PAGE of pooled leaf samples (n=8) showing accumulation of HFB fusion proteins (expected size indicated by arrows) in Nicotiana benthamiana. (B) Immunoblot analysis with anti StrepII-tag antibody indicates some degradation of the fusion proteins. Equal amounts of total soluble protein were loaded on all gels. A leaf infiltrated with only a construct for P19 was used as a negative control.
