# Supplementary material for: Novel Hydrophobin Fusion Tags for Plant-Produced Fusion Proteins
Source: PLoS One. 2016 Oct 5;11(10):e0164032. doi: 10.1371/journal.pone.0164032 (PMC5051927; doi:10.1371/journal.pone.0164032)
Supplement: S4 Fig — (A) Coomassie stained SDS-PAGE gels of representative samples from ATPS (Fig 1B). Lane numbering refers to: 1) TSP; 2) residue; 3) recovered phase. (B) Concentrations of fusion proteins in residue and recovered phase in comparison to initial concentration in plant leaf extract (TSP). Error bars indicate standard deviation (n = 4). (PDF) [file pone.0164032.s004.pdf]

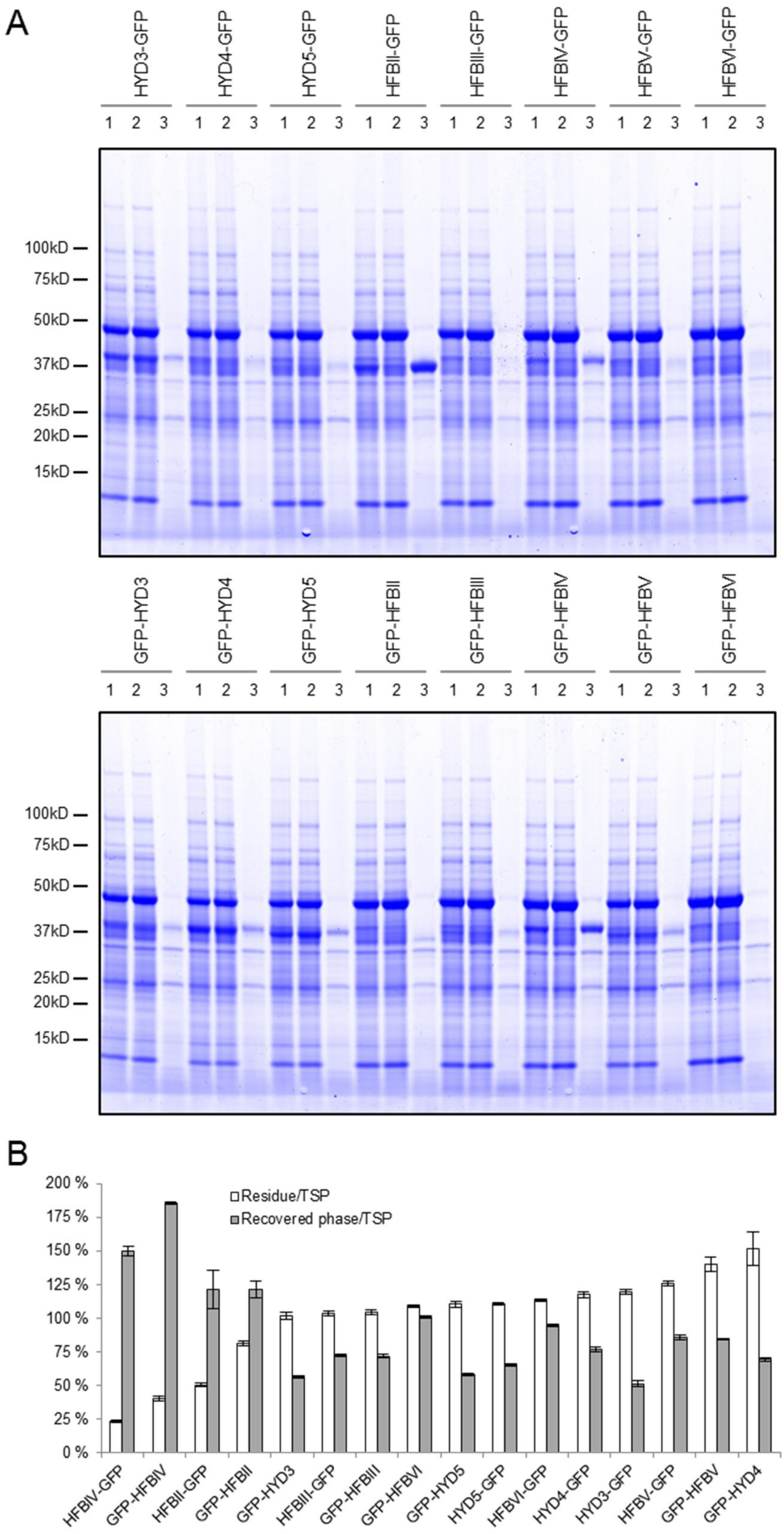

S4 Fig. SDS-PAGE illustrating aqueous two phase separation. (A) Coomassie stained SDS-PAGE gels of representative samples from ATPS (Fig. 1B). Lane numbering refers to: 1) TSP; 2) residue; 3) recovered phase. (B) Concentrations of fusion proteins in residue and recovered phase in comparison to initial concentration in plant leaf extract (TSP). Error bars indicate standard deviation (n=4).
