# Supplementary material for: Novel Hydrophobin Fusion Tags for Plant-Produced Fusion Proteins
Source: PLoS One. 2016 Oct 5;11(10):e0164032. doi: 10.1371/journal.pone.0164032 (PMC5051927; doi:10.1371/journal.pone.0164032)
Supplement: S6 Fig — (A) Coomassie stained SDS-PAGE and (B) an immunoblot of pooled samples (n = 3) from starting solution, residue phase and recovered phase in ATPS comparing HFBI-GFP, HFBII-GFP and HFBIV-GFP (Fig 3B). Detection was performed with anti-c-Myc tag primary antibody (rabbit, A00172, GeneScript) and a secondary antibody for IR-detection (goat anti-rabbit, IR Dye® 680RD, LI-COR Biosciences, Germany). (PDF) [file pone.0164032.s006.pdf]

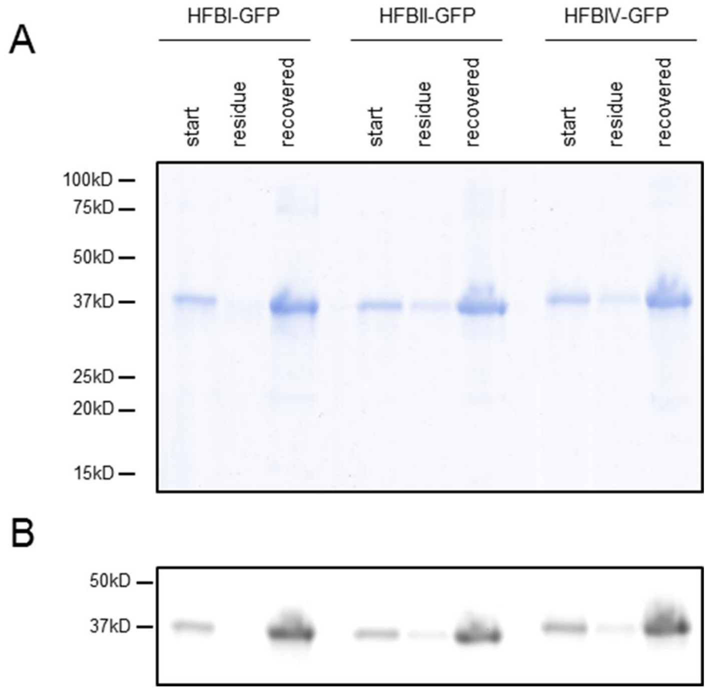

S6 Fig. SDS-PAGE and immunoblot illustrating aqueous two-phase separation with purified proteins. (A) Coomassie stained SDS-PAGE and (B) an immunoblot of pooled samples (n=3) from starting solution, residue phase and recovered phase in ATPS comparing HFBI-GFP, HFBII-GFP and HFBIV-GFP (Fig. 3B). Detection was performed with anti-c-Myc tag primary antibody (rabbit, A00172, GeneScript) and a secondary antibody for IR-detection (goat anti-rabbit, IR Dye® 680RD, LI-COR Biosciences, Germany).
