# Supplementary material for: Novel Hydrophobin Fusion Tags for Plant-Produced Fusion Proteins
Source: PLoS One. 2016 Oct 5;11(10):e0164032. doi: 10.1371/journal.pone.0164032 (PMC5051927; doi:10.1371/journal.pone.0164032)
Supplement: S1 Table — The sequence coding for the extended N-terminal part of HFBVI, presumably a cell wall binding domain (amino acids 1–179), was not included in the coding sequence. (PDF) [file pone.0164032.s007.pdf]

S1 Table. Genbak ID numbers for original gene sequences and Uniprot ID numbers for amino acid sequences used in this study. The sequence coding for the extended N-terminal part of HFBVI, presumably a cell wall binding domain (amino acids 1-179), was not included in the coding sequence.

Protein	Nucleotide (NCBI*)	Peptide (Uniprot**)	Reference
HFBI	Z68124.1	P52754	[1]
HFBII	Y11894.1	P79073	[2]
HFBIII	18483774	G0RVE5	[3]
HFBIV	18481287	G0RHN0	[4]
HFBV	18480804	G0RBZ9	this study
HFBVI	18481131	G0RFI5	this study
HYD3	AY155498	Q6YF30	[5]
HYD4	AY155499	Q6YF29	[5]
HYD5	AY158024	Q6YD93	[5]

* http://www.ncbi.nlm.nih.gov/ ** http://www.uniprot.org/

[1] Nakari-Setälä, T., Aro, N., Kalkkinen, N., Alatalo, E., et al., Genetic and biochemical characterization of the Trichoderma reesei hydrophobin HFBI. *Eur. J. Biochem.* 1996, *235*, 248–255.

[2] Nakari-Setala, T., Aro, N., IlmeN, M., Munoz, G., et al., Differential Expression of the Vegetative and Spore-Bound Hydrophobins of Trichoderma Reesei Cloning and Characterization of the Hfb2 Gene. *Eur. J. Biochem.* 1997, *248*, 415–423.

[3] Kisko, K., Szilvay, G.R., Vuorimaa, E., Lemmetyinen, H., et al., Self-assembled films of hydrophobin protein HFBIII from Trichoderma reesei. *J. Appl. Crystallogr.* 2007, *40*, 355–360.

[4] Espino-Rammer, L., Ribitsch, D., Przylucka, A., Marold, A., et al., Two novel class II hydrophobins from Trichoderma spp. stimulate enzymatic hydrolysis of poly(ethylene terephthalate) when expressed as fusion proteins. *Appl. Environ. Microbiol.* 2013, *79*, 4230–8.

[5] Fuchs, U., Czymmek, K.J., Sweigard, J.A., Five hydrophobin genes in Fusarium verticillioides include two required for microconidial chain formation. *Fungal Genet. Biol.* 2004, *41*, 852–64.
